# Aging increases proprioceptive error for a broad range of movement speed and distance estimates in the upper limb

**DOI:** 10.3389/fnhum.2023.1217105

**Published:** 2023-10-11

**Authors:** Duncan Thibodeau Tulimieri, Jennifer A. Semrau

**Affiliations:** ^1^Biomechanics and Movement Science (BIOMS), University of Delaware, Newark, DE, United States; ^2^Department of Kinesiology and Applied Physiology, University of Delaware, Newark, DE, United States; ^3^Department of Biomedical Engineering, University of Delaware, Newark, DE, United States

**Keywords:** proprioception, aging, robotics, sensorimotor control, upper limb

## Abstract

Previous work has identified age-related declines in proprioception within a narrow range of limb movements. It is unclear whether these declines are consistent across a broad range of movement characteristics that more closely represent daily living. Here we aim to characterize upper limb error in younger and older adults across a range of movement speeds and distances. The objective of this study was to determine how proprioceptive matching accuracy changes as a function of movement speed and distance, as well as understand the effects of aging on these accuracies. We used an upper limb robotic test of proprioception to vary the speed and distance of movement in two groups: younger (*n* = 20, 24.25 ± 3.34 years) and older adults (*n* = 21, 63 ± 10.74 years). The robot moved one arm and the participant was instructed to mirror-match the movement with their opposite arm. Participants matched seven different movement speeds (0.1–0.4 m/s) and five distances (7.5–17.5 cm) over 350 trials. Spatial (e.g., End Point Error) and temporal (e.g., Peak Speed Ratio) outcomes were used to quantify proprioceptive accuracy. Regardless of the speed or distance of movement, we found that older controls had significantly reduced proprioceptive matching accuracy compared to younger control participants (*p* ≤ 0.05). When movement speed was varied, we observed that errors in proprioceptive matching estimates of spatial and temporal measures were significantly higher for older adults for all but the slowest tested speed (0.1 m/s) for the majority of parameters. When movement distance was varied, we observed that errors in proprioceptive matching estimates were significantly higher for all distances, except for the longest distance (17.5 cm) for older adults compared to younger adults. We found that the magnitude of proprioceptive matching errors was dependent on the characteristics of the reference movement, and that these errors scaled increasingly with age. Our results suggest that aging significantly negatively impacts proprioceptive matching accuracy and that proprioceptive matching errors made by both groups lies along a continuum that depends on movement characteristics and that these errors are amplified due to the typical aging process.

## Introduction

Coordinated movement is necessary for humans to interact with their environment effortlessly and efficiently. Proprioception, the sense of our body’s location and motion (Sherrington, 1907), is critical for movement planning (Sarlegna and Sainburg, 2009), error estimation (Jones et al., 2010), and error correction (Sainburg et al., 1995; Gosselin-Kessiby et al., 2009; Shadmehr et al., 2010; Scott, 2016). Previous work has shown that not only can disease or injury negatively impact upper limb proprioception (Carey et al., 1996; Konczak et al., 2007; Dukelow et al., 2010; Semrau et al., 2013, 2015; Simo et al., 2014; Gurari et al., 2018), but typical aging has also been found to negatively impact proprioceptive function (Stelmach and Sirica, 1986; Adamo et al., 2007; Herter et al., 2014). Overall, diminished proprioceptive function of the upper limb can lead to poor coordination, general “clumsiness,” and significant potential for injury due to a lack of awareness of one’s body in space.

Previous work in the upper limb that has found proprioceptive decline or impairment with age or stroke has typically examined proprioceptive accuracy within a narrow range of movement speeds and movement distances; typically, one movement speed and/or one movement distance (Adamo et al., 2009; Dukelow et al., 2010, 2012; Semrau et al., 2013, 2015, 2017, 2018; Herter et al., 2014, 2019; Kenzie et al., 2014, 2017; Li and Wu, 2014; Contu et al., 2017; Acosta-Sojo and Martin, 2021). While these studies have added to our understanding of how age and stroke affects proprioceptive behavior, they have done so at a limited range of movements. There have been three studies that have assessed proprioception and modulated movement distance (Stelmach and Sirica, 1986; Adamo et al., 2007; Boisgontier and Swinnen, 2015). These studies have revealed that proprioceptive matching errors increase as movement distance increases. In the lower limb, specifically the ankle, work assessing proprioceptive matching behavior at self-selected and fast speeds found that error increased in the fast speed condition, especially in older adults (Boisgontier and Nougier, 2013). Other techniques besides bilateral mirror-matching, namely psychophysics, that are used for determining proprioceptive thresholds typically utilize movements at a variety of speeds and/or distances to find the “threshold,” or the smallest/slowest possibly detectable movement (Kokmen et al., 1978; Carey et al., 1996; Wycherley et al., 2005; Wright et al., 2011; Ingemanson et al., 2016; Rinderknecht et al., 2017; Lowrey et al., 2020). While these studies shed light on the minimum stimulus required for proprioceptive detection, they do not inform us as to whether certain movement characteristics (e.g., speed and/or distance) have “preferred” status in the performance of naturalistic movements.

The movements that we make on a daily basis are widely varied in both speed and distance of movement, thus making it critical to understand how the proprioceptive system responds and reproduces stimuli from a wide range of behavior. Therefore, the main goal of this study was to determine the influence of movement speed and movement distance on upper limb proprioceptive accuracy in younger and older adults. We hypothesized that aging would negatively impact proprioceptive accuracy and predicted that older adults would have increased proprioceptive errors compared to younger adults. Secondly, we predicted that we would observe larger differences in speed estimation between older and younger controls (Goble and Brown, 2009). Third, we predicted that older adults would make larger proprioceptive errors that scale with movement distance (Stelmach and Sirica, 1986; Adamo et al., 2007). Lastly, we aimed to examine interactions between movement speed and movement distance to determine if proprioceptive error was influenced by the combination of movement speed and movement distance.

## Materials and methods

A total of 41 participants (younger controls: *N* = 20 (24.25 ± 3.34 years, Range: [19–30 years], 7 males/13 females) and older controls: *N* = 21 (63 ± 10.74 years, Range: [45–79 years], 9 males/12 females)) were included in the study. The following inclusion criteria were used for all participants: 18 years or older and having normal or corrected-to-normal vision. The following exclusion criteria were used for all participants: prior history of neurological disease or injury (e.g., Parkinson’s Disease, Traumatic Brain Injury), previous history of significant upper body injury (e.g., rotator cuff tear), or history of a disease that may impact sensation (e.g., peripheral neuropathy). All participants completed the Edinburgh Handedness Inventory to determine hand dominance (Oldfield, 1971). Informed consent was obtained from all individual participants included in the study.

### Experimental apparatus

**Figure 1:**
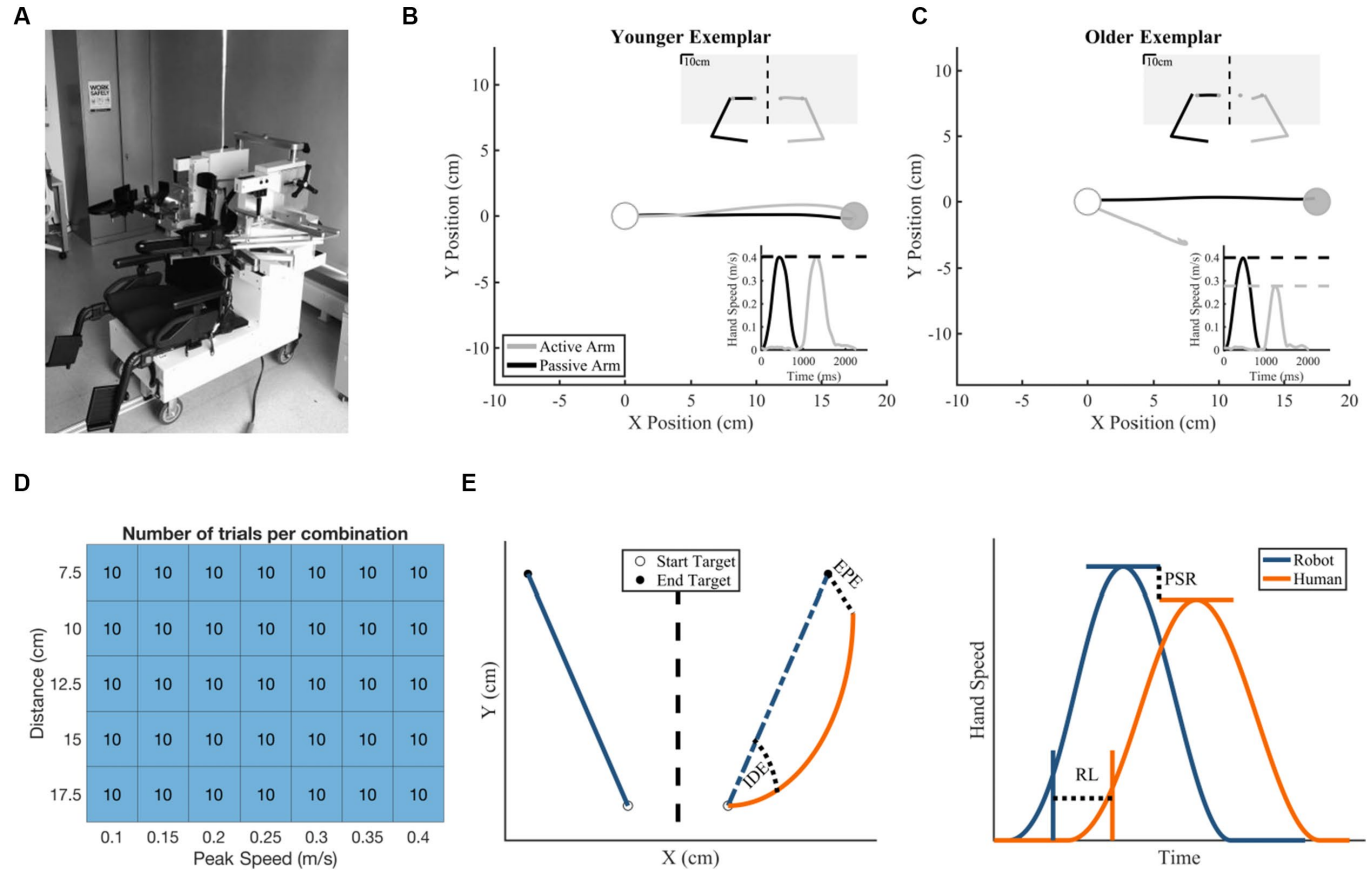
Robotic exoskeleton, exemplar subject data, and methods information. **(A)** KINARM Exoskeleton Robot. **(B,C)** Hand trajectory with insets of hand speed (bottom right) and total workspace view (upper right). The white target is the start target and the gray target is the end target. **(B)** Exemplar younger control behavior with small Initial Direction Error (2.78°), near matched Peak Speed Ratio (1.01 active/passive), small End Point Error (0.736 cm), and short Response Latency (959 ms). **(C)** Exemplar older control behavior (Initial Direction Error = 20.6°, Peak Speed Ratio = 0.693 active/passive, End Point Error = 10.83 cm, Response Latency = 934 ms). **(D)** Number of matching movements made per speed and distance combination by each participant. **(E)** Schematic of temporal [End Point Error (EPE) and Initial Direction Error (IDE)] and spatial [Response Latency (RL) and Peak Speed Ratio (PSR)] proprioceptive outcomes.

The KINARM Exoskeleton Lab (BKIN Technologies, Kingston, ON, Canada) was used to collect kinematic data of the upper limbs (Figure 1A; Scott, 1999). Participants were seated in the robotic exoskeleton with their shoulders at ~85° of abduction and their upper and lower arm secured within arm troughs. The proximal and distal segments of the robot were adjusted to each participants’ respective limb proportions. Once the exoskeleton was fit, participants were wheeled into the horizontally-mounted virtual reality display with their head resting on a padded fabric affixed to the virtual reality display. Participants had two degrees of freedom within the robot: horizontal flexion/extension at the shoulder and flexion/extension at the elbow. The exoskeleton is capable of producing mechanical loads at both the shoulder and elbow joints to passively move the participant’s arm with a bell-shaped velocity profile given a desired location and duration. A calibration procedure preceded each participants’ experiment to create the most accurate virtual reality display. Vision of the arms was occluded with a metal shutter and a bib.

### Experimental protocol

The robot passively moved one arm (passive arm) and participants were instructed to mirror-match the movement with their opposite arm (active arm) without the use of visual feedback. Specifically, individuals were instructed to mirror-match the speed, direction, and length of each movement with their active arm as soon as they perceived their passive arm being moved (Supplementary material; Figures 1B,C). For the purposes of experimental protocol, a trial began when the robot started to passively move one arm and ended when the participant verbally indicated they were matched, and the robot operator clicked a button. The button click revealed vision of the hands’ current positions (1 cm white cursor at fingertip of both hands) and the desired positions (2 cm cyan circles mirrored across body midline), and participants were then instructed to move their active arm (white cursor) into the cyan circle in order to move to the next trial. After their active hand was in the target for a randomized amount of time (400–1,000 ms), all visual information was extinguished, and the next trial began. The purpose of this visual feedback was to ensure that all participants began each trial with both limbs in a mirror-matched position. No data from time periods with visual feedback was used in subsequent analyses. The passive arm (i.e., arm moved by robot) was counterbalanced within each group to account for effects of handedness. In the younger (≤30 years old) control group, 10 participants had their dominant arm passively moved by the robot and 10 participants had their non-dominant arm passively moved by the robot. In the older (≥45 years old) control group, 10 participants had their dominant arm passively moved by the robot and 11 participants had their non-dominant arm passively moved by the robot.

We assessed five distances and seven peak speeds with a total of 10 reaches per combination of peak speed and distance, resulting in a total of 350 reaches to 350 targets within a 20 × 20 cm workspace (Figure 1D). The assessed distances ranged from 7.5 to 17.5 cm with steps of 2.5 cm, for a total of 5 distances tested. The assessed peak speeds ranged from 0.1 to 0.4 m/s with steps of 0.05 m/s, for a total of 7 speeds tested. Together, there were a total of 35 speed x distance combinations. Each passive robotic movement had a desired movement profile, initialized with a randomized start and end target. The angle of this movement was randomly chosen from a uniform distribution from 0° to 359° with consideration of workspace boundaries. Within these two targets, movement speed was commanded to generate the passive movement implemented via a bell-shaped velocity profile. A custom MATLAB algorithm was designed to create a schedule of target locations and displacement durations, such that (1) speed and distance combinations were randomly ordered (2) each speed and distance combination was sampled equally, and (3) direction of the movement was random. For this experiment, the same randomized schedule of target locations and displacement durations were used for all participants.

### Kinematic data analysis

The data were analyzed to determine the magnitude of spatial and temporal proprioceptive matching errors. Data was initially parsed from the time of trial start (passive movement begins) to end of trial (participant indicates they are matched/operator button click), which is prior to when vision of hands and targets is revealed. Peak speed was defined as the maximum hand speed within this time frame. Movement onset, for each arm, was defined as the time when hand speed was greater than or equal to 10% of the respective peak hand speed for 50 consecutive milliseconds. Movement onset of the active arm was additionally required to be at least 150 ms after the movement onset of the passive arm to ensure movements were not anticipated. Movement end, for each arm, was defined as the time when the respective hand speed decreased below 10% of the respective peak speed for 50 consecutive milliseconds or when the trial ended, whichever occurred first. All analyses and figures were done using custom MATLAB code.

### Spatial parameters

Kinematic data of the active limb in the x-dimension was mirrored (sign-flipped) across the workspace to make direct comparisons between passive and active movements (Figure 1E). End Point Error was calculated as the Euclidean distance between the passive limb and the active limb at their respective movement end locations (Polit and Bizzi, 1979; Messier et al., 2003; Sarlegna and Sainburg, 2009; Dukelow et al., 2010) (Equation 1). A larger End Point Error indicates that the active arm was farther away from the “ideal” end point of the passive arm. Initial Direction Error was calculated as the angle between vectors connecting the start location and the locations at peak speed for the passive arm and the mirrored active arm (Sarlegna and Sainburg, 2009; Semrau et al., 2013) (Equation 2). A larger Initial Direction Error indicates that the direction of movement of the active arm was greater than the “ideal” trajectory of the passive arm.


(1) $$\mathrm{End}\;\mathrm{Point}\;\mathrm{Error}=\sqrt{\sum {\left(\mathrm{Offse}t_{\mathrm{passive}}-\mathrm{Offse}t_{\mathrm{active}}\right)}^{2}}$$

Equation 1. End Point Error. Offset is defined as the hand position during movement offset. Movement offset was calculated as the first time the hand speed fell below 10% of the peak speed or the trial ended. The offset of the active arm was reflected across the x-axis for the calculation.


(2) $$\begin{array}{l} \mathrm{Initial}\;\mathrm{Direction}\;\mathrm{Error}\\ =\cos^{-1}\left(\frac{\mathrm{Initial}\;\mathrm{Movement}\;\mathrm{Vecto}r_{\mathrm{passive}}}{\mathrm{Initial}\;\mathrm{Movement}\;\mathrm{Vecto}r_{\mathrm{active}}}\right) \end{array}$$

Equation 2. Initial Direction Error. Initial movement vectors were determined for both the passive and active arms. This vector began at the starting x,y position and stopped at the x,y position when peak speed occurred. Initial Direction Error was then calculated by determining the angular difference between the two vectors.

### Temporal parameters

Response Latency was calculated as the difference in time between movement onset of the passive arm and movement onset of the active arm (Vercher et al., 1997; Semrau et al., 2013) (Equation 3). A larger Response Latency indicates that the participant took longer to initiate their movement in response to the passive movement of the robot. Peak Speed Ratio was calculated as the quotient of peak speed of the active arm and peak speed of the passive arm (Semrau et al., 2013) (Equation 4). A Peak Speed Ratio greater than 1 indicates that the active arm had a greater peak speed than the passive arm.


(3) $$\mathrm{Response}\;\mathrm{Latency}=\mathrm{Onse}t_{\mathrm{active}}-\mathrm{Onse}t_{\mathrm{passive}}$$

Equation 3. Response Latency. Movement onset was calculated as the first time the hand speed fell below 10% of the peak speed when looking backwards in time from the time of peak speed.


(4) $$\mathrm{Peak}\;\mathrm{Speed}\;\mathrm{Ratio}=\frac{\mathrm{Peak}\;\mathrm{spee}d_{\mathrm{active}}}{\mathrm{Peak}\;\mathrm{spee}d_{\mathrm{passive}}}$$

Equation 4. Peak Speed Ratio. Peak speed of the arm was defined as the peak of the hand speed between movement onset and movement offset. Movement onset was defined as in Response Latency (Equation 3). Movement offset for Peak Speed Ratio was defined the same as in End Point Error (Equation 1).

### Statistical analyses

To analyze group differences, permutation tests and common language effect size (CLES) were utilized (Good, 2005). Directional permutation tests (*H*_0_: *younger controls* ≥ *older controls*) were used to compare between groups for the following parameters: End Point Error, Initial Direction Error, and Response Latency, and to statistically confirm age differences between the older control group and the younger control group. Non-directional permutation tests (*H*_0_: *younger controls* = *older controls*) were used for comparisons between groups for the fourth (Peak Speed Ratio) proprioceptive matching outcome. Non-directional tests were used for Peak Speed Ratio because error increased as the value deviated from 1 in either (positive or negative) direction. All permutation testing was completed by using averaged parameter (e.g., Response Latency) data from individual subjects, shuffling group assignments, and implementing 1,000,000 iterations to compare the resultant distribution to test for differences in the averages of the resampled groups. CLES values were calculated exact (i.e., permute all possible combinations). This method was used to examine (1) group level performance without consideration of speed or distance, (2) group level differences for speed within a single parameter (e.g., Response Latency), (3) group level differences for distance within a single parameter. For example, in examining effects of speed on Initial Direction Error performance, for each of the seven speed values tested [0.1, 0.15, 0.2, 0.25, 0.3, 0.35, 0.4 m/s], a permutation test was utilized to determine if the error distribution of Initial Direction Error was significantly greater for older controls compared to younger controls. This process was then repeated for all parameters (End Point Error, Initial Direction Error, Response Latency, and Peak Speed Ratio) described above for each of the seven speed values and each of the five distance values.

To examine overall patterns of proprioceptive matching error between groups for differences in speed or distance, for each parameter (e.g., Response Latency), we fit individual participants averaged data (5 data points for distance analyses, 7 data points for speed analyses) to a line using ordinary least squares. This yielded an intercept ( $\beta_{0}$ ) and slope ( $\beta_{1}$ ) coefficient for each participant for each proprioceptive matching outcome to examine parameter-based differences between groups for error magnitude (intercept) and error scaling (slope). To determine if these distributions were significantly different between groups, we used permutation tests to compare the distributions of intercept and slope coefficients between groups for each proprioceptive matching outcome by speed/distance. Lastly, we utilized a two-way ANOVA with averaged group data to quantify interaction effects between speed and distance within each group for each proprioceptive matching parameter (alpha = 0.05).

## Results

We assessed proprioceptive matching accuracy over a broad range of speeds and distances in neurologically intact younger and older controls using a bilateral proprioceptive matching task. On average, our younger control group was significantly younger than our older control group (*p* < 0.001, CLES = 100). There were 18 right-handed and 2 left-handed younger controls and all older controls were right-handed.

### Overall proprioceptive error

**Figure 2:**
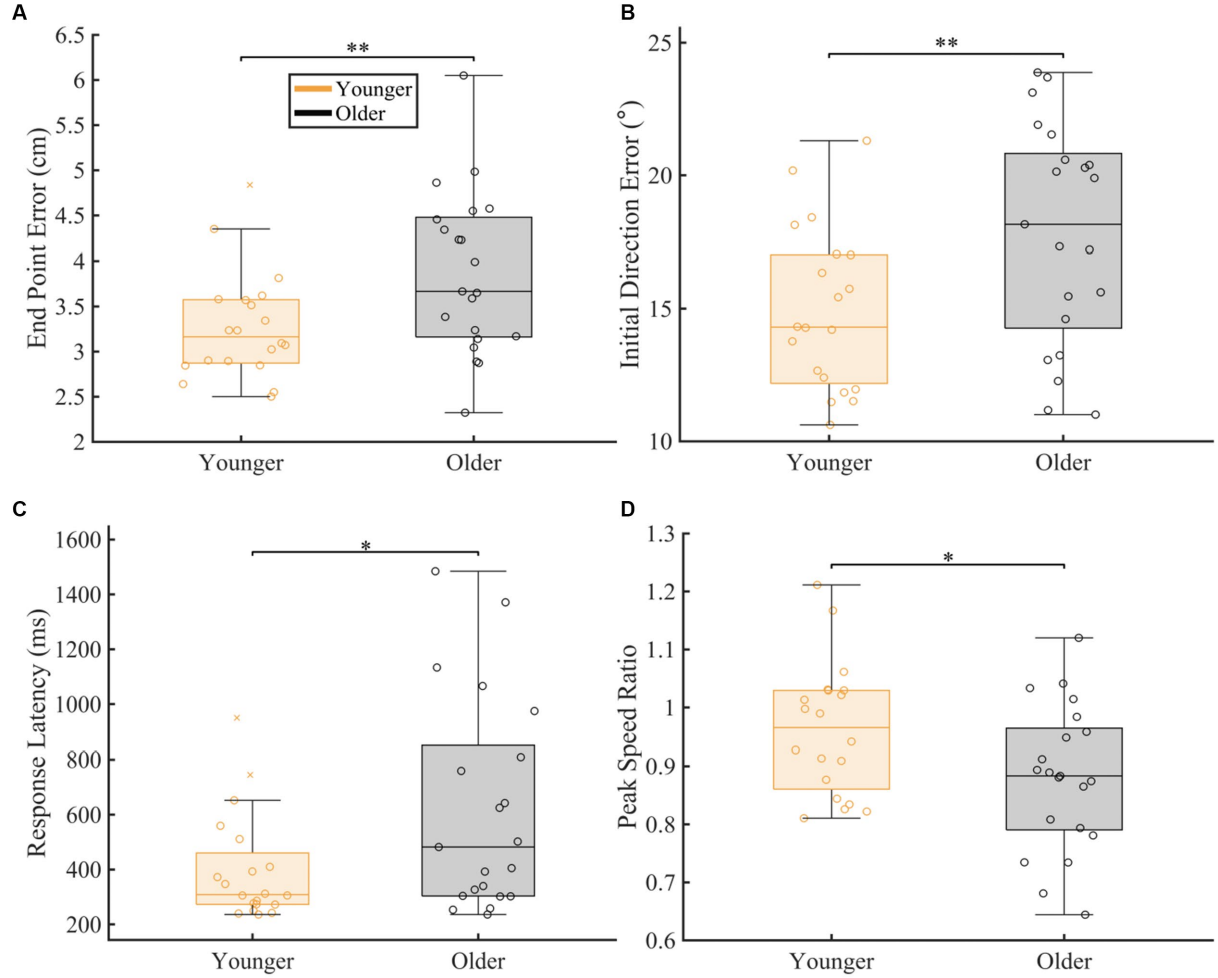
Overall group comparison of spatial and temporal proprioceptive measures. The box charts display median, lower and upper quartiles, minimum and maximum that are not outliers. The colored circles indicate subject level averages. The number of asterisks’ represent the respective *p*-values for the permutation test: *p ≤ 0.05, **p ≤ 0.01, ***p ≤ 0.001. The magnitude of difference was calculated using common language effect sizes (CLES). **(A–D)** Comparison of spatial [**(A)** End Point Error (*p* = 0.007, CLES = 71.90) and **(B)** Initial Direction Error (*p* = 0.008, CLES = 70.95)] and temporal [**(C)** Response Latency (*p* = 0.013, CLES = 67.38) and **(D)** Peak speed ratio (*p* = 0.031, CLES = 67.62)] proprioceptive measures between younger controls and older controls.

To compare overall proprioceptive matching accuracy between our groups, we compared group performance for each proprioceptive matching measure, regardless of speed and distance (Figure 2). We found significant differences between groups for all our spatial (End Point Error and Initial Direction Error) and temporal (Response Latency and Peak Speed Ratio) proprioceptive matching measures. Specifically, older adults had significantly higher End Point Error (*p* = 0.007, CLES = 71.90), Initial Direction Error (*p* = 0.008, CLES = 70.95), and Response Latency (*p* = 0.013, CLES = 67.38) and less accurate Peak Speed Ratio (*p* = 0.031, CLES = 67.62) compared to younger adults.

### Proprioceptive differences as a function of robot movement speed

**Figure 3:**
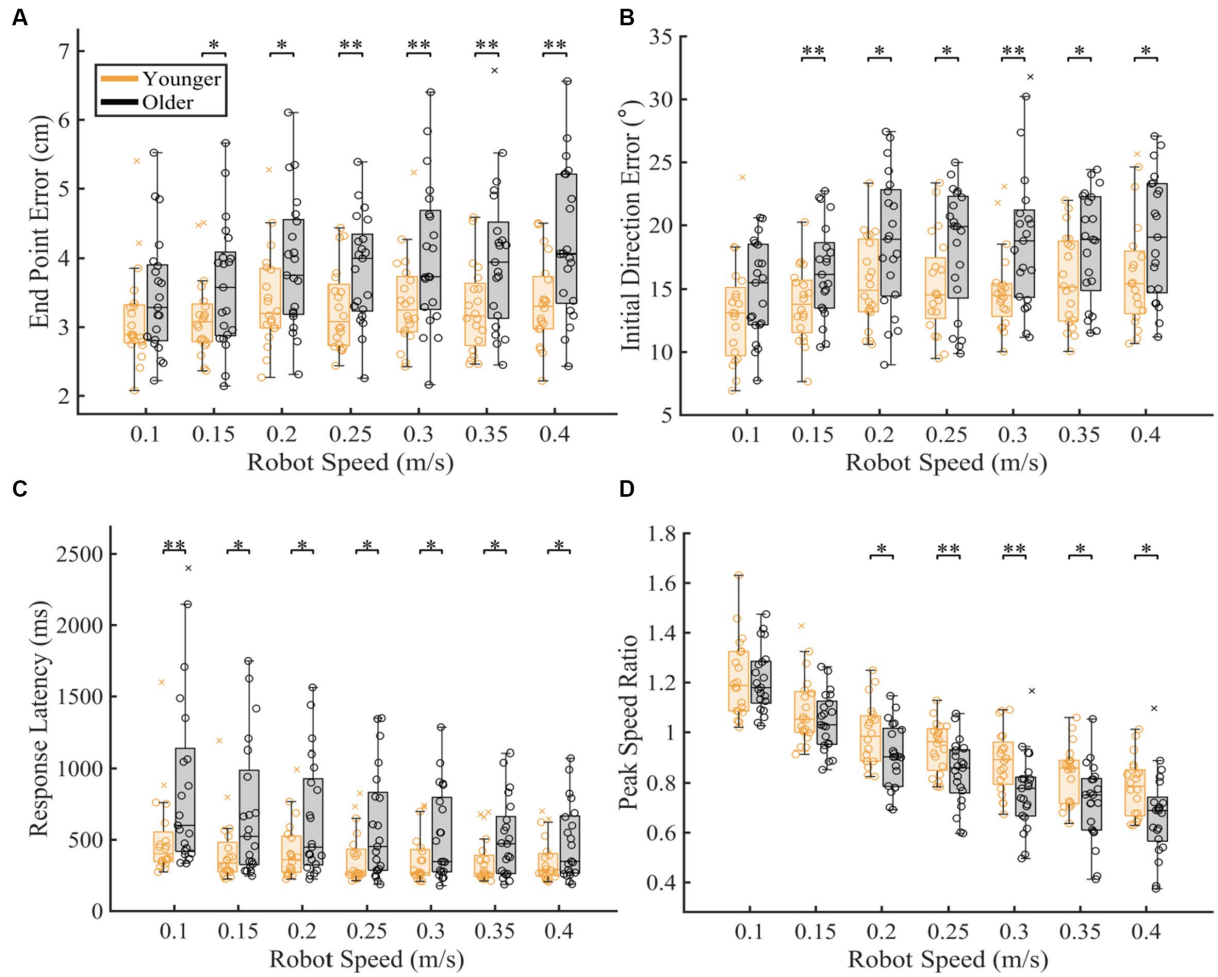
Proprioceptive accuracy within each robot speed. The box charts at each speed display median, lower and upper quartiles, minimum and maximum that are not outliers. Colored circles represent subject level averages for each outcome at each speed. Significance bars and asterisks represent the respective p-values from a permutation test: *p ≤ 0.05 and **p ≤ 0.01. **(A–D)** Younger and older controls average **(A)** End Point Error, **(B)** Initial Direction Error, **(C)** Response Latency, and **(D)** Peak Speed Ratio within all tested speed. *P*-values and CLES for each comparison can be found in Table 1.

To understand which movement speeds were contributing to the differences seen in overall proprioceptive matching error, we examined the difference in group performance within each speed tested (Figure 3). We found that older adults had significantly worse proprioception compared to younger controls for End Point Error [*p* < 0.05 for 0.15, 0.20, 0.25, 0.30, 0.35, 0.40 m/s], Initial Direction Error [*p* < 0.05 for 0.15, 0.20, 0.25, 0.30, 0.35, 0.40 m/s], Response Latency [*p* < 0.05 for 0.10, 0.15, 0.20, 0.25, 0.30, 0.35, 0.40 m/s], and Peak Speed Ratio [*p* < 0.05 for 0.20, 0.25, 0.30, 0.35, 0.40 m/s] (Full statistical results included in Table 1).

**Table 1:** Statistics for within speed and distance comparisons between younger and older adults.

	Parameter			
	End Point Error (cm)	Initial Direction Error (deg)	Response Latency (ms)	Peak Speed Ratio
Speed (m/s)				
0.10	CLES = 60.71; *p* = 0.101	CLES = 63.81; *p* = 0.080	CLES = 72.62; *p* = 0.009**	CLES = 51.19; *p* = 0.864
0.15	CLES = 65.24; *p* = 0.032*	CLES = 71.67; *p* = 0.008**	CLES = 69.52; *p* = 0.011*	CLES = 57.14; *p* = 0.252
0.20	CLES = 67.14; *p* = 0.029*	CLES = 68.81; *p* = 0.014*	CLES = 66.43; *p* = 0.017*	CLES = 66.19; *p* = 0.039*
0.25	CLES = 72.14; *p* = 0.006**	CLES = 68.33; *p* = 0.020*	CLES = 68.10; *p* = 0.011*	CLES = 72.62; *p* = 0.008**
0.30	CLES = 70.00; *p* = 0.009**	CLES = 72.62; *p* = 0.003**	CLES = 66.67; *p* = 0.022*	CLES = 74.05; *p* = 0.009**
0.35	CLES = 71.43; *p* = 0.004**	CLES = 69.29; *p* = 0.021*	CLES = 68.57; *p* = 0.015*	CLES = 70.48; *p* = 0.015*
0.40	CLES = 74.05; *p* = 0.002**	CLES = 67.14; *p* = 0.025*	CLES = 63.81; *p* = 0.030*	CLES = 70.48; *p* = 0.020*
Distance (cm)				
7.5	CLES = 75.48; *p* = 0.002**	CLES = 72.62; *p* = 0.002**	CLES = 65.95; *p* = 0.011*	CLES = 69.76; *p* = 0.017*
10.0	CLES = 70.00; *p* = 0.011*	CLES = 70.95; *p* = 0.006**	CLES = 68.10; *p* = 0.010*	CLES = 68.57; *p* = 0.021*
12.5	CLES = 68.81; *p* = 0.028*	CLES = 66.67; *p* = 0.021*	CLES = 71.19; *p* = 0.009**	CLES = 65.24; *p* = 0.053
15.0	CLES = 70.95; *p* = 0.010**	CLES = 64.76; *p* = 0.046*	CLES = 65.95; *p* = 0.018*	CLES = 67.14; *p* = 0.041*
17.5	CLES = 68.10; *p* = 0.012*	CLES = 62.62; *p* = 0.066	CLES = 67.86; *p* = 0.019*	CLES = 61.67; *p* = 0.152

CLES: common language effect size. Stars represent significance level: *p ≤ 0.05, **p ≤ 0.01, ***p ≤ 0.001.

**Figure 4:**
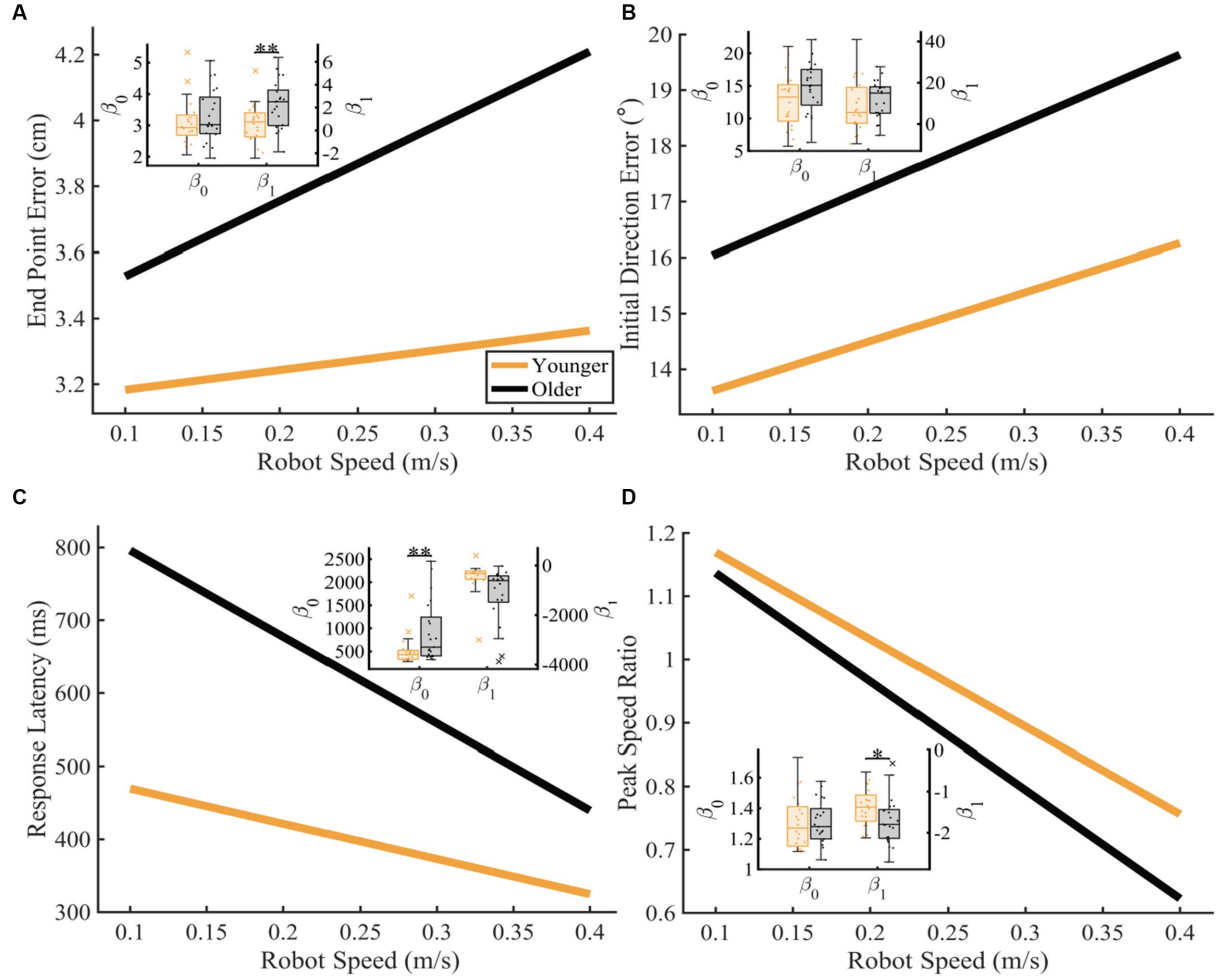
Proprioceptive accuracy as a function of robot speed. Thick lines represent the average of the group’s parameter (intercept and slope) distributions. Group parameter distributions can be found in the inset where the left *y*-axis represents the intercept distributions, and the right *y*-axis represents the slope distributions. Permutation tests were used to compare fit parameters and CLES was used to determine magnitude of difference. **(A–D)** Younger and older controls average **(A)** End Point Error (younger: *y* = 3.12 + 0.60x and older: *y* = 3.30 + 2.27x; intercept: *p* = 0.243, CLES = 56.67; slope: *p* = 0.004, CLES = 74.29), **(B)** Initial Direction Error (younger: *y* = 12.73 + 8.81x and older: *y* = 14.83 + 12.01x; intercept: *p* = 0.051, CLES = 65.24; slope: *p* = 0.178, CLES = 60.48), **(C)** Response Latency (younger: *y* = 517.98 + −483.35x and older: *y* = 914.59 + −1187.40x; intercept: *p* = 0.009, CLES = 72.86; slope: *p* = 0.991, CLES = 76.90), and **(D)** Peak Speed Ratio (younger: *y* = 1.31 + −1.38x and older: *y* = 1.31 + −1.71x; intercept: *p* = 0.984, CLES = 54.29; slope: *p* = 0.047, CLES = 69.76) as a function of robot speed. Significance bars and asterisks represent the respective *p*-values from a permutation test: *p ≤ 0.05 and **p ≤ 0.01.

To further understand the influence of age on proprioceptive matching accuracy as function of speed, we examined how each proprioceptive matching outcome changed as a function of speed (Figure 4). When we examined the intercept ( $\beta_{0}$ ) and slope ( $\beta_{1}$ ) coefficients of the proprioceptive matching outcomes by speed, we found that for End Point Error and Peak Speed Ratio, older controls had significantly greater (End Point Error: *p* = 0.005, CLES = 74.29) and significantly different (Peak Speed Ratio: *p* = 0.047, CLES = 69.76) slopes compared to younger controls. We also found that for Response Latency, intercept values for older controls were significantly greater than younger controls indicating a higher degree of error for older adults (Response Latency: *p* = 0.009, CLES = 72.86). This shows that for End Point Error and Peak Speed Ratio, older adults had increased error as a function of passive movement speed. Additionally, for Response Latency, older adults had a higher level of proprioceptive matching error regardless of movement speed. We also examined the influence of handedness for task performance for younger and older adults. Here, we separated those individuals where the robot moved the dominant arm (Younger Adults: *N* = 10, Older Adults: *N* = 10) and where the robot moved the non-dominant arm (Younger Adults: *N* = 10, Older Adults: *N* = 11) into two groups. We found that across the different reference speeds tested, younger adults’ dominant arm showed slight improvement in proprioceptive matching error for End Point Error [*p* < 0.05 for 0.10, 0.15, 0.20, 0.25, and 0.30 m/s, and intercept] and Initial Direction Error [*p* < 0.05 for 0.10, 0.15, 0.20, and 0.25 m/s, and intercept and slope] compared to their non-dominant arm. In contrast, we found no significant differences within our older adult group for dominant vs. non-dominant limbs.

### Proprioceptive differences as a function of robot movement distance

**Figure 5:**
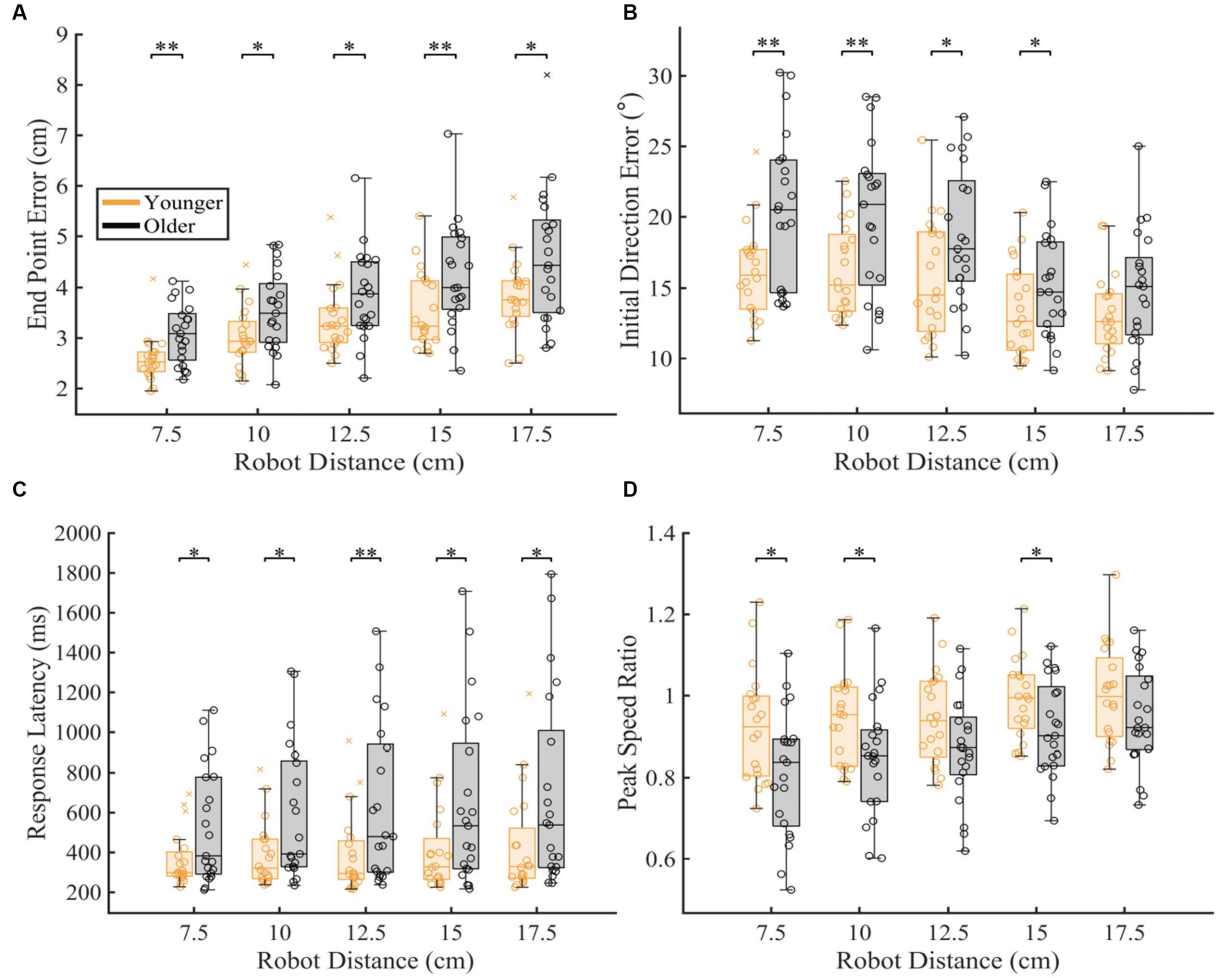
Proprioceptive accuracy within each robot distance. The box charts at each speed display median, lower and upper quartiles, minimum and maximum that are not outliers. Colored circles represent subject level averages for each outcome at each distance. Significance bars and asterisks represent the respective p-values from a permutation test: *p ≤ 0.05 and **p ≤ 0.01. **(A–D)** Younger and older controls average **(A)** End Point Error, **(B)** Initial Direction Error, **(C)** Response Latency, and **(D)** Peak Speed Ratio within all tested distances. *P*-values and CLES for each comparison can be found in Table 1.

To understand which distances were contributing to differences seen in overall proprioceptive matching error, we examined the difference in group performance within each distance tested (Figure 5). We found that older adults had significantly worse End Point Error [*p* < 0.05 for 7.5, 10.0, 12.5, 15.0, 17.5 cm], Initial Direction Error [*p* < 0.05 for 7.5, 10.0, 12.5, 15 cm], Response Latency [*p* < 0.05 for 7.5, 10.0, 12.5, 15.0, 17.5 cm] and significantly different Peak Speed Ratio [*p* < 0.05 for 7.5, 10.0, 15 cm] at a majority of distances for these measures when compared to younger controls (Table 1).

**Figure 6:**
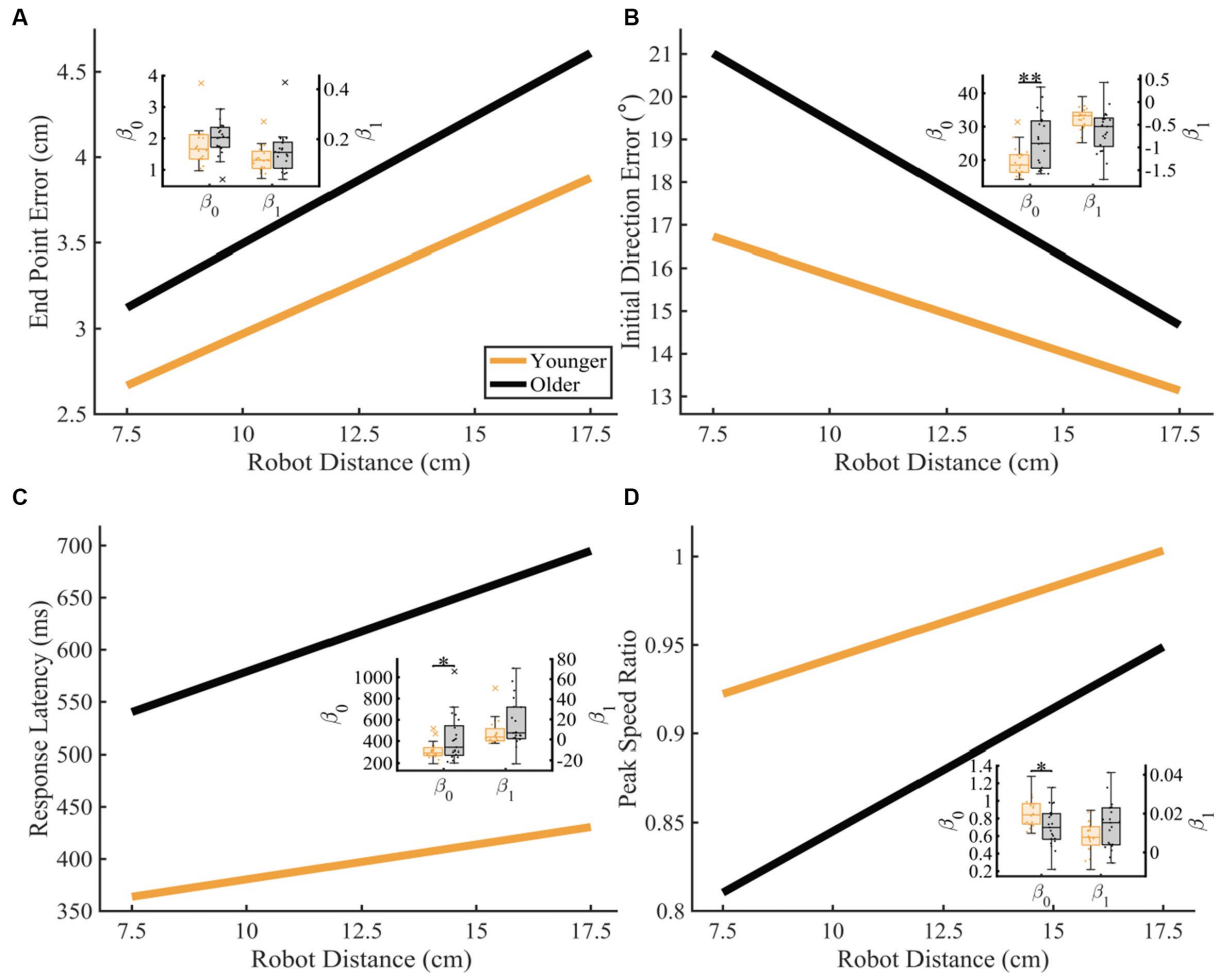
Proprioceptive accuracy as a function of robot distance. Thick lines represent the average of the group’s parameter (intercept and slope) distributions. Group parameter distributions can be found in the inset where the left *y*-axis represents the intercept distributions, and the right *y*-axis represents the slope distributions. Permutation tests were used to compare fit parameters and CLES was used to determine magnitude of difference. **(A–D)** Younger and older adults average **(A)** End Point Error (younger: *y* = 1.76 + 0.12x and older: *y* = 2.01 + 0.15x; intercept: *p* = 0.089, CLES = 67.62; slope: *p* = 0.110, CLES = 61.67), **(B)** Initial Direction Error (younger: *y* = 19.42 + −0.36x and older: *y* = 25.77 + −0.63x; intercept: *p* = 0.002, CLES = 74.29; slope: *p* = 0.988, CLES = 70.48), **(C)** Response Latency (younger: *y* = 313.45 + 6.70x and older: *y* = 424.12 + 15.48x; intercept: *p* = 0.014, CLES = 63.57; slope: *p* = 0.076, CLES = 64.05), and **(D)** Peak Speed Ratio (younger: *y* = 0.86 + 0.01x and older: *y* = 0.71 + 0.01x; intercept: *p* = 0.015, CLES = 70.71; slope: *p* = 0.082, CLES = 63.81) as a function of robot distance. Significance bars and asterisks represent the respective *p*-values from a permutation test: *p ≤ 0.05 and **p ≤ 0.01.

To further understand how proprioceptive matching accuracy changed as a function of distance, we examined the intercept and slope coefficients of the proprioceptive matching outcomes by individual distance (Figure 6). We found that for older adults, Initial Direction Error, Response Latency, and Peak Speed Ratio (Initial Direction Error: *p* = 0.002, CLES = 74.29, Response Latency: *p* = 0.014, CLES = 63.57, Peak Speed Ratio: *p* = 0.016, CLES = 70.71) had intercepts that indicated greater overall error across distances compared to younger controls. This suggests that for multiple parameters, older adults had a higher error magnitude compared to younger controls. As described above, to examine effects of handedness, groups were split into individuals where the robot moved the dominant arm and where the robot moved the non-dominant arm. We found that across the different reference distances tested, younger adults’ dominant arm showed slight improvement in proprioceptive matching error for End Point Error [*p* < 0.05 for 12.5, 15.0, and 17.5 cm, and slope], Initial Direction Error [*p* < 0.05 for 12.5 and 15.0 cm], and Path Length Ratio [*p* < 0.05 for intercept and slope] compared to their non-dominant arm. In contrast, we found no significant differences within our older adult group for dominant vs. non-dominant limbs.

### Interaction of speed and distance on proprioceptive measures

To gain an understanding of the interaction of speed and distance on proprioceptive matching accuracy, we examined the interaction term of a two-way ANOVA on each groups average data. We found that younger controls had a significant speed x distance interaction effect for Peak Speed Ratio (*F* = 7.080, *p* = 0.012), while older adults had a significant speed x distance interaction effect for Response Latency (*F* = 16.901, *p* < 0.001).

## 4. Discussion

We found that aging had significant effects on proprioceptive accuracy, and that age-related increases in proprioceptive error often depended on the characteristics of the reference movement (i.e., speed or distance). We found that older adults generally had higher levels of proprioceptive error across all distances, and that this error response scaled similarly in younger controls (Figures 5, 6). Notably, we found that while older adults generally had more proprioceptive error across different reference speeds, the amount of proprioceptive error scaled differently for changes in reference speed compared to younger adults (Figures 4A,D). This was particularly robust when the reference movement was drawn from the faster end of the distribution of tested speeds. Lastly, we found minimal interaction effects for speed and distance of the reference movement for both groups, suggesting that error magnitude was not necessarily reliant on a combination of speed and distance parameters.

### Proprioceptive accuracy is dependent on speed and distance of the reference movement

Previous work has detailed the presence of age-related declines in proprioception (Kokmen et al., 1978; Stelmach and Sirica, 1986; Adamo et al., 2007; Wright et al., 2011; Ingemanson et al., 2016; Rinderknecht et al., 2017; Acosta-Sojo and Martin, 2021). This work, and others, has typically examined participants’ ability to replicate movement speed or distance (e.g., reference movement of 20 degree elbow flexion) (Adamo et al., 2007, 2009; Wingert et al., 2009; Dukelow et al., 2010; Semrau et al., 2013; Li and Wu, 2014; Contu et al., 2017; Watkins et al., 2020; Acosta-Sojo and Martin, 2021) and has found that aging-related increases in proprioception are common across different testing mechanisms including bilateral proprioceptive testing (Stelmach and Sirica, 1986; Adamo et al., 2007, 2009; Kalisch et al., 2012; Herter et al., 2014; Acosta-Sojo and Martin, 2021) and unilateral or within arm proprioceptive testing (Ferrell et al., 1992; Adamo et al., 2007, 2009; Wright et al., 2011; Wingert et al., 2014; Ingemanson et al., 2016; Rinderknecht et al., 2017; Acosta-Sojo and Martin, 2021). However, it is important to note that these studies, as well as our own previous work (Semrau et al., 2013), have generally used methods that test a relatively narrow range of movement types. This has provided limited interpretation on whether the proprioceptive system is sensitive to particular movement characteristics or whether it exhibits a similar level of error across all movement characteristics.

In the current study, we have used methodology that allows us to examine proprioceptive error responses that consider a broad range of movement types, ranging from short, slow reference movements to long, fast reference movements (see Methods). Here, we have used this method to understand two main aspects of proprioception: (1) whether the magnitude of proprioceptive error changes as a function of the characteristics of the reference movement, and (2) whether aging negatively impacts proprioceptive estimations and if so, are proprioceptive error patterns different for younger and older adults. Importantly, we first observed that older adults show considerable decline in nearly all proprioceptive parameters compared to younger adults and that when we examine specific speeds and distances, these differences are consistent. Secondly, we find that while overall patterns of error are significantly higher for older adults compared to younger adults, there were also significant differences in proprioceptive error scaling for changes in reference speed for older adults. Here, we found that for End Point Error, older adults scaled their error more rapidly as reference speed increased, as indicated by a much steeper slope (Figure 4A). Additionally, we observed that older adults were able to similarly match movement speed when the reference movement was slow (0.1 m/s), but as the reference speed increased, the magnitude of speed matching error as measured by the Peak Speed Ratio parameter also increased, as demonstrated by older adults greatly underestimating the reference speed at the fastest reference movements. In contrast, while we observed overall higher levels of proprioceptive error when the reference movement changed distance, we did not observe differences in error scaling for changes in distance as we did for changes in speed (Figure 6). Work by Goble and Brown (2009) examined upper limb proprioceptive matching behavior at two different reference speeds (30°/s and 60°/s) in younger adults. They found that proprioceptive matching errors were significantly greater when participants experienced the faster reference speed. They concluded that this increase in proprioceptive error at the faster speed was due to sensory attenuation that occurs during the movement. Previous work from Collins et al. (1998) found that faster movements of the wrist resulted in a greater amount of sensory attenuation, as demonstrated by significant reductions in muscular sense (i.e., twitch amplitude). We believe that our current results demonstrate similar sensory attenuation at faster movement speeds for older adults, and that the differences observed between younger adults and older adults demonstrate an amplification of this attenuation as a result of the aging process. However, we must note that various control properties of motor units change with aging, such as decreased average firing rate (Erim et al., 1999) and decreases in the number of motor units (Brown et al., 1988), which may contribute to other changes in limb perception with age, such as the sense of effort (Monjo et al., 2018).

### Mechanisms of age-related proprioceptive decline

While we expected to observe that older adults had higher levels of proprioceptive error compared to younger adults, we did not expect that differences in error patterns relative to speed or distance to be so robust. These differences suggest that as we age, the distribution of our proprioceptive accuracy or sensitivity narrows. There are several contributing factors that may explain these differences. The first is that during normal aging, neuroanatomical changes occur. Here, there is a loss of white matter (Guttmann et al., 1998) over time and diminished white matter integrity has been shown to be predictive of poor proprioception and balance in the older adults (Van Impe et al., 2012); however the impact on upper limb proprioception is unknown (Zhai et al., 2020). In contrast, volumetric changes have not been observed in sensorimotor cortical areas or callosal projections to these areas in older adults (Raz et al., 2004; Michielse et al., 2010). This suggests that aged-related changes to fibers responsible for carrying afferent information may play a role in increases in proprioceptive error. Second, as we age, there is loss of both extrafusal and intrafusal muscle fibers (Brooks and Faulkner, 1994). Previous studies have found that, with age, the number of muscle spindles decrease in number over time and receive less innervation than in “young” muscle (Swallow, 1966; Swash and Fox, 1972; Proske and Gandevia, 2012; Landelle et al., 2021). It is reasonable to suspect that diminished function of sensory receptors leads to more variable proprioceptive estimation. Lastly, it is possible that the increased errors and age-related differences in proprioceptive error patterning that we observe in older adults may be due to decrements in sensorimotor integration that occur with age (Brown et al., 2018; Sorrentino et al., 2021), that may result in higher proprioceptive gains or a noisier system that is less sensitive to changes in perceived body position or movement. A contributing factor to this may be from sensory attenuation that occurs as a result of age. A recent study has suggested that sensory attenuation leads to alterations in the internal estimation of force for older adults (Parthasharathy et al., 2022). While the authors report that they did not observe declines in static proprioception in older adults, they found that older adults significantly over-estimated self-produced forces. This result, in combination with previous work finding increased sensory attenuation during both slow and fast passive movements (Collins et al., 1998), suggests that alterations in afferent signaling from the muscles as well as efference copy signals contribute to diminished proprioceptive accuracy in older adults.

### Limitations

The paradigm used to assess proprioception in this study is not without limitations. One limitation of this paradigm is the inherent motor component of the matched movements. If a participant had a motor deficit in their matching arm, one could confuse the deficits seen in their data as sensory deficits, when in fact they were motor deficits. In this study, we counterbalanced the arm moved by the robot in each of our groups in order to ensure that any interlimb differences in proprioception were accounted for and any sensory declines related to aging would not potentially be masked by improved ability using the dominant limb. When we examined inter-limb differences, we found some differences for when the robot moved the dominant vs. non-dominant arm, but only in younger adults. These results are in line with previous work describing minimal differences in task performance with a bilateral matching task, such as the one we use here (Goble and Brown, 2009), suggesting that the small differences seen in the younger adult group require further exploration to determine if these are true differences in proprioception or variability in performance. Another limitation is the range of movement speeds and distances tested. We set movement speed and distance bounds to fit the robot’s mechanical capacity as well as participants physiological capacity. We could have tested with more granularity between the bounds, but this would have increased the task time beyond a comfortable time for many participants.

## Conclusion

We found that older age was significantly associated with increased amounts of proprioceptive error in a bilateral proprioceptive matching task. In older adults and younger adults, we found that proprioceptive error scaled as a function of both speed and distance, with faster speeds and longer distances typically resulting in larger amounts of proprioceptive error for both groups. Further, when testing different reference speeds, we found that for some parameters in older adults, proprioceptive error scaled differently, suggesting that not only is proprioceptive accuracy diminished in older adults, but that error responses can change as a function of age. These results have significant implications for how we think about proprioceptive testing in older adults and understanding how proprioception is affected as a function of age, particularly related to how sensory signals are impacted as a result of the aging process.

## Data availability statement

The raw data supporting the conclusions of this article will be made available by the authors, without undue reservation.

## Ethics statement

The studies involving humans were approved by the University of Delaware Institutional Review Board. The studies were conducted in accordance with the local legislation and institutional requirements. The participants provided their written informed consent to participate in this study.

## Author contributions

DT was involved with study implementation, participant recruitment, data analysis, drafting, and editing of the manuscript. JS was the primary investigator for the current study and was involved in study conception and design, participant recruitment, data analysis, and writing of the manuscript. The first draft of the manuscript was written by DT. JS commented on all versions of the manuscript. All authors read and approved the final manuscript.
