# Supplementary material for: Aging increases proprioceptive error for a broad range of movement speed and distance estimates in the upper limb
Source: Front Hum Neurosci. 2023 Oct 11;17:1217105. doi: 10.3389/fnhum.2023.1217105 (PMC10598783; doi:10.3389/fnhum.2023.1217105)
Supplement: Supplementary file 1 [file Presentation_1.pptx]

## Slide 1
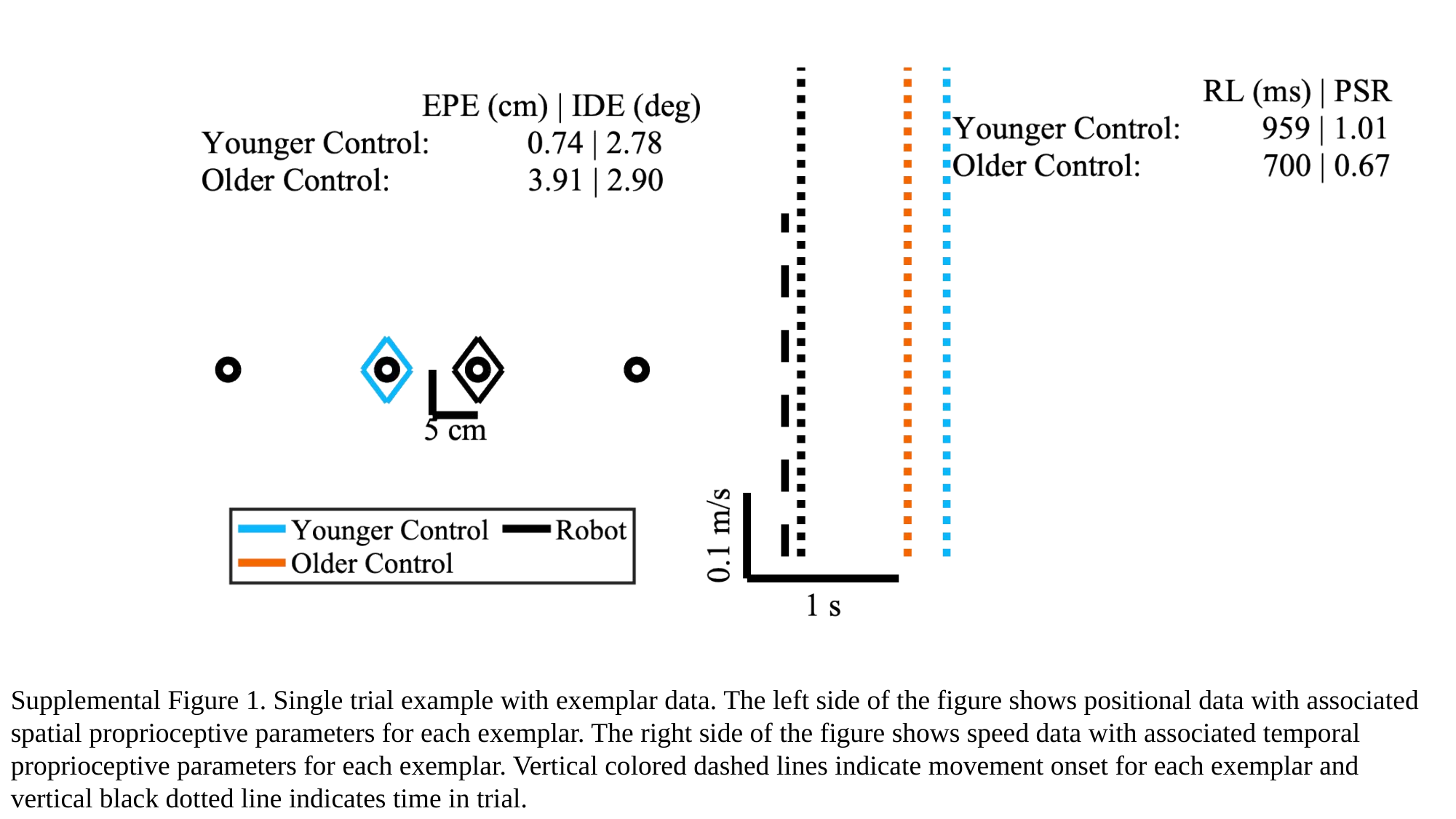

Supplemental Figure 1. Single trial example with exemplar data. The left side of the figure shows positional data with associated spatial proprioceptive parameters for each exemplar. The right side of the figure shows speed data with associated temporal proprioceptive parameters for each exemplar. Vertical colored dashed lines indicate movement onset for each exemplar and vertical black dotted line indicates time in trial.
